# CNN–Aided Optical Fiber Distributed Acoustic Sensing for Early Detection of Red Palm Weevil: A Field Experiment †

**DOI:** 10.3390/s22176491

**Published:** 2022-08-29

**Authors:** Islam Ashry, Biwei Wang, Yuan Mao, Mohammed Sait, Yujian Guo, Yousef Al-Fehaid, Abdulmoneim Al-Shawaf, Tien Khee Ng, Boon S. Ooi

**Affiliations:** 1 Computer, Electrical and Mathematical Sciences and Engineering (CEMSE) Division, King Abdullah University of Science and Technology (KAUST), Thuwal 23955-6900, Saudi Arabia; 2 Department of Electronic and Information Engineering, The Hong Kong Polytechnic University, Hong Kong SAR, China; 3 Zhongshan Institute of Changchun University of Science and Technology, Zhongshan 528400, China; 4 Center of Date Palms and Dates, Ministry of Environment, Water and Agriculture, Al-Hassa 31982, Saudi Arabia

**Keywords:** red palm weevil, optical fiber distributed acoustic sensing, machine learning

## Abstract

Red palm weevil (RPW) is a harmful pest that destroys many date, coconut, and oil palm plantations worldwide. It is not difficult to apply curative methods to trees infested with RPW; however, the early detection of RPW remains a major challenge, especially on large farms. In a controlled environment and an outdoor farm, we report on the integration of optical fiber distributed acoustic sensing (DAS) and machine learning (ML) for the early detection of true weevil larvae less than three weeks old. Specifically, temporal and spectral data recorded with the DAS system and processed by applying a 100–800 Hz filter are used to train convolutional neural network (CNN) models, which distinguish between “infested” and “healthy” signals with a classification accuracy of ∼97%. In addition, a strict ML-based classification approach is introduced to improve the false alarm performance metric of the system by ∼20%. In a controlled environment experiment, we find that the highest infestation alarm count of infested and healthy trees to be 1131 and 22, respectively, highlighting our system’s ability to distinguish between the infested and healthy trees. On an outdoor farm, in contrast, the acoustic noise produced by wind is a major source of false alarm generation in our system. The best performance of our sensor is obtained when wind speeds are less than 9 mph. In a representative experiment, when wind speeds are less than 9 mph outdoor, the highest infestation alarm count of infested and healthy trees are recorded to be 1622 and 94, respectively.

## 1. Introduction

The red palm weevil (RPW) Rhynchophorus ferrugineus (Olivier) is one of the world’s major invasive pest species that attacks date, coconut, ornamental, and oil palms in a variety of agricultural ecosystems worldwide [1,2]. In the past four decades, the RPW has spread rapidly and has been detected in more than 60 countries in the Mediterranean, North Africa, the Middle East, and parts of the Caribbean and Central America [1,3]. This plague has a significant social and economic impact on the date palm industry and the livelihoods of farmers in the affected areas [4,5]. The RPW causes economic losses estimated at millions of USD annually, whether through lost production or pest control costs. In Italy, Spain, and France, for example, the control of the RPW and losses are expected to reach about $235 million by 2023, unless a strict containment program is implemented [1].

Treatment of RPW-infested trees by chemical injection [6], for example, is a straightforward and effective method; however, the detection of the RPW threat at an early stage is challenging. Since the RPW larvae feed internally in tree trunks, they are difficult to detect in palm groves before the tree shows visible signs of distress in a well-advanced infestation stage, when the tree is difficult to save by treatment [7]. In the literature, sniffing dogs [8], electronic nose [9], X-ray-based tomography [10], and thermal imaging [11] show promising results for the early detection of RPW; however, they lack feasibility on large farms due to their slow scanning processes. For large-scale implementation, in contrast, the most promising early detection methods rely on acoustic sensors that identify the gnawing sounds of RPW larvae while they are chewing on the core of a palm trunk [12,13,14]. Current acoustic detection methods implant acoustic probes into individual tree trunks and construct a wireless network to communicate with the sensors [13]. Existing acoustic detection methods suffer from the following drawbacks: (1) assigning an acoustic probe to each tree is not cost-effective, especially for a large farm with hundreds of trees, (2) the detection provides point sensing at the location of inserting the acoustic probe. In other words, the sensor cannot monitor the entire tree trunk, with the same sensitivity, (3) acoustic probes are invasive and may damage trees or create nests for insects.

For the purpose of early detection of RPW, we recently introduced the use of an optical fiber distributed acoustic sensor (DAS), designed using the phase-sensitive optical time-domain reflectometer (Φ-OTDR) [12,15,16]. The original approach is described in [12], where, starting at a DAS interrogation unit, a single optical fiber cable is extended and wound non-invasively around tree trunks to possibly monitor a vast farm in a short time. Compared to the point sensing offered by the acoustic probes, the optical fiber DAS can provide distributed monitoring of many trees and also along the trunk of each tree. However, in [12], the distinction between healthy and infected trees is based on a simple signal processing method (signal-to-noise ratio (SNR) measurement), which is difficult to rely on in an outdoor farm with different noise sources. Thus, in [15], we then presented the use of neural network-based machine learning (ML) algorithms as powerful tools for classifying healthy and infested trees, using the data recorded by an optical fiber DAS. However, the latter work was carried out in a laboratory environment using an artificial sound of RPW larvae, produced by a loudspeaker implanted within a tree. Finally, in [16], we extended our aforementioned work to use the ML-assisted optical fiber DAS to detect true weevil larvae in a well-controlled environment.

**Figure 1:**
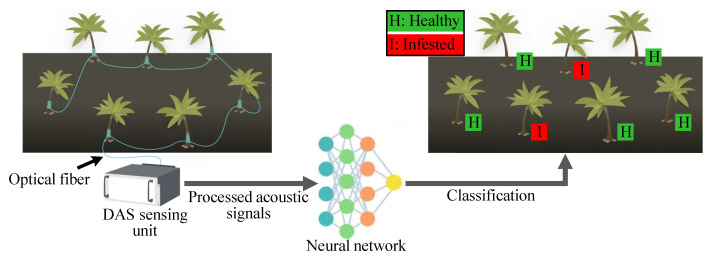
Overall approach for RPW detection using an optical fiber DAS and machine learning.

Here, we substantially extend our aforementioned work to use a convolutional neural network (CNN)-aided optical fiber DAS to recognize healthy and truly RPW-infested trees in an outdoor farm. The overall sensing approach is presented in Figure 1, where the optical fiber DAS unit records and processes acoustic signals from individual trees on the farm. Then, the processed data are passed to the trained CNN model that distinguishes healthy and infested trees. Training, validation, and testing of the CNN model are performed using acoustic temporal/spectral “infested” signals (from trees infested with 2–3-week-old RPW larvae) and “healthy signals" (from healthy trees placed in calm or noisy environment). Additionally, we discuss the limitations of using the designed sensor outdoors. To the best of our knowledge, no such deployment of ML-assisted optical fiber DAS for RPW detection in an outdoor farm has been previously conducted. Integrating ML with optical fiber DAS to detect the true sound of RPW larvae, especially in outdoor farms, would be very useful for controlling the spread of RPW infestation, and this work adds an important step toward designing a practical RPW detection sensor.

## 2. Experimental Setup

**Figure 2:**
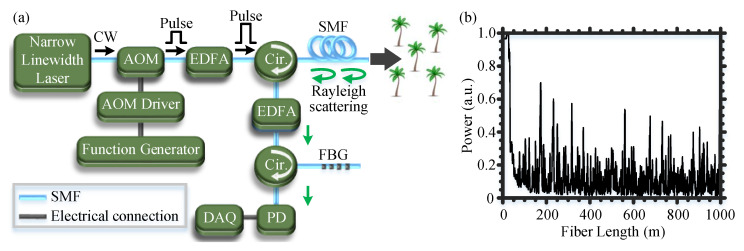
(**a**) Experimental setup of the Φ-OTDR-based optical fiber DAS used for the detection of RPW. Cir.: circulator. (**b**) A representative Rayleigh trace recorded by the optical fiber DAS along a 1-km SMF.

The Φ-OTDR-based optical fiber DAS used for the detection of RPW is schematically shown in Figure 2a [17], where a narrow linewidth laser produces a continuous wave (CW) light of a 1550-nm wavelength, a 40-mW optical power, and a 100-Hz linewidth. Using an acousto-optic modulator (AOM), the CW light is modulated into optical pulses of a 5-kHz repetition rate and a 50-ns width (∼5-m spatial resolution DAS). Next, the optical pulses are amplified with an erbium-doped fiber amplifier (EDFA) and then injected through a circulator into a standard single-mode fiber (SMF) of a ∼1-km length. The SMF is extended throughout the farm, and we loop a ∼5-m fiber section around each tree trunk. We further add a layer of plastic wrap over the fiber section to reinforce the fiber attachment to the tree and to mitigate the impact of the environmental acoustic noise. The backscattered Rayleigh signal from the SMF is directed via the circulator toward another EDFA for power amplification, and the amplified spontaneous emission (ASE) noise of the EDFA is discarded using a fiber Bragg grating (FBG). Finally, the filtered Rayleigh signal is detected by a photodetector (PD) and sampled by a digitizer. The design of the used optical fiber DAS system is conventional, which was initially described in [18,19]. However, the combination of the DAS system with ML for the early detection of RPW outdoors is new and significantly beneficial.

Figure 2b shows an example of a Rayleigh trace recorded along the ∼1-km SMF. The high-power signal found at the beginning of the SMF is common and is caused by the Fresnel reflection from the front facet of the SMF. In ideal scenarios when the refractive index is unperturbed along the optical fiber, the subsequent temporal Rayleigh traces along the fiber should be identical [17,20]. Thus, the differential signal between the subsequent temporal Rayleigh traces and an initial reference one should ideally be zero along the entire fiber. In the case that weevil larvae are chewing on a tree trunk, their eating sound perturbs the refractive index of the SMF, which yields to altering the Rayleigh intensity only at the site of the infested tree. Applying the normalized differential method [21] and the fast Fourier transform (FFT) to the subsequent Rayleigh traces, the temporal and spectral acoustic signals along the optical fiber can be calculated, respectively.

## 3. Classifying “Infested” and “Healthy” Acoustic Signals Using CNNs

In general, neural networks can provide high efficiency in image classification [22]. Recently, additional advanced methods such as integrating principal component analysis (PCA) and local binary pattern (LBP) [23], and mathematical morphology spectrum entropy [24] are used to improve the accuracy and generalization ability of hyperspectral image classification and signal feature extraction, respectively. It was found that CNN architectures can handle a large amount of data, similar to that produced by the optical fiber DAS, and at the same time can reveal patterns associated with the larvae eating sound [15]. In this section, we compare the efficiencies of classifying “infested” and “healthy” acoustic signals when using the DAS temporal and spectral data as separate inputs to CNN architectures. To reduce the sensor false alarm rate, in addition, we present an approach for integrating the classification results generated when using the temporal and spectral data.

In terms of data organization and labeling for the CNN architectures, the spatial sampling of the digitizer used is ∼0.5 m and we wind a ∼5-m fiber section around each tree trunk; thus, the fiber around each tree trunk is represented by 10 spatial points. For each spatial point on the tree trunk, a digitizer reading lasts for a 100-ms period, which is 500 temporal measurements because the pulse repetition rate is 5 kHz. Since CNNs have been proven to be highly effective in classifying images [22], we organize the temporal data into a 2D matrix (10 spatial points × 500 temporal measurements). Similarly, the spectral data are organized as a (10 spatial points × 250 spectral components) 2D matrix, obtained by applying the FFT to the temporal data of each spatial point.

**Figure 3:**
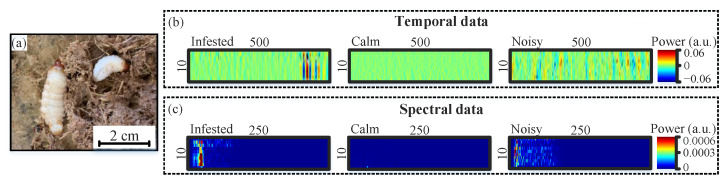
(**a**) Weevil larvae less than three weeks old. Representative examples of the temporal “infested”, “calm”, and ”noisy” images (**b**), and their corresponding spectral images (**c**).

During the CNN training process, we rely on supervised learning such that the data are labeled based on the tree condition (infested or healthy) and the SNR value of the temporal acoustic signal at the location of the tree. The “infested” data are recorded from six artificially infested trees with weevil larvae less than three weeks old (Figure 3a), which is considered to be an early stage of infestation [12]. A detailed description of the artificial infestation process and age control of weevil larvae is provided in [12]. To ensure that the recorded acoustic “infested” signals are caused by the larvae, we place the artificially infested trees in a well-controlled environment so that the trees are not exposed to major acoustic noise such as that produced by outdoor wind [15]. Under these conditions for the infested trees, if the SNR is greater than 2 dB (the minimum acceptable SNR for optical fiber DAS [21]), we label and record the signal as “infested”. On the other hand, the ”healthy” data are collected from 10 healthy trees, of which six are on an outdoor farm that includes typical sources of acoustic noise produced by wind, birds, humans, etc. and the other four healthy trees are in the above-mentioned controlled environment. We divide the ”healthy” data as ”calm” and ”noisy” signals, where the SNR is <2 dB and >2 dB, respectively.

In total for the CNN architecture associated with the temporal/spectral data, we record 18,000 examples of the “infested” signals and another 18,000 examples (9000 “calm” and 9000 “noisy”) of the “healthy” signals. To evaluate the performance of the CNN architectures, the recorded temporal/spectral examples are split as 60% (21,600 examples) training, 20% (7200 examples) validation, and 20% (7200 examples) testing datasets. All of the examples are processed by applying a [100–800 Hz] band-pass filter. This filter mitigates the environmental acoustic noise that typically has low frequencies, less than 100 Hz, and discards the high-frequency (larger than 800 Hz) noise produced by the electronic/optical components in the DAS system, without affecting the dominant weevil larvae acoustic frequencies [12,15]. Figure 3b,c shows representative examples of the input images for the CNN models when using the “infested”, “calm”, and ”noisy” temporal data and their corresponding spectral images, respectively.

**Figure 4:**
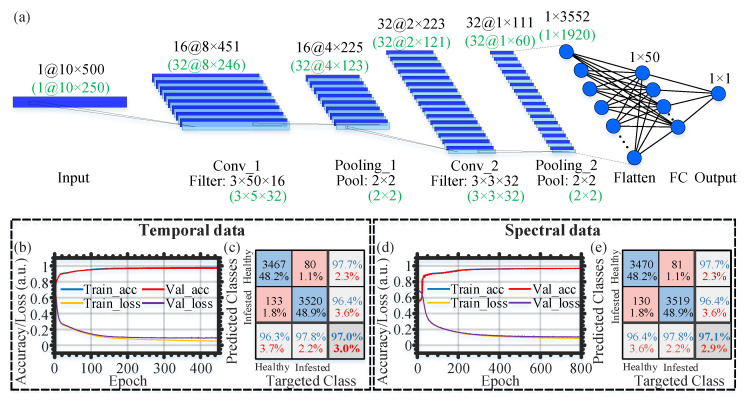
(**a**) The CNN architecture for classifying “infested” and “healthy” temporal (spectral) signals. Conv: convolutional; FC: fully-connected; The dimensions of the CNN architecture associated with the spectral data are written in green. Training and validation history (**b**,**d**) and confusion matrix (**c**,**e**) when using the temporal/spectral data.

Figure 4a shows the architecture of the CNN model used to handle the temporal (spectral) input data. The CNN architecture includes an input layer, two pairs of convolutional and max pooling layers, a flattened layer, a fully-connected layer, and an output layer, respectively. The first convolutional layer has the ReLu activation function and comprises 16 (32) filters of a 3 × 50 (3 × 5) size and a 1 × 1 (1 × 1) stride, while the first max pooling layer has a 2 × 2 (2 × 2) pool size. In contrast, the second convolutional layer also has the ReLu activation function and includes 32 (32) filters of a 3 × 3 (3 × 3) size and a 1 × 1 (1 × 1) stride, while the second max pooling layer includes a 2 × 2 (2 × 2) pool size. Following the flattened layer, the fully-connected layer has the ReLU activation function and includes 50 (50) nodes. Eventually, the output layer of the CNN contains a single node with a sigmoid activation function for binary classification (“infested” or “healthy” signal).

The adopted CNN model, shown in Figure 4a, contains many structural configuration features and parameters for the training process. The setting of the structure and parameters are very flexible, and there is no universal rule among different tasks. We follow the standard practice and use the classification accuracy as the primary evaluation standard to try different parameters repeatedly until the performance stops improving. For instance, about the number of interlayers in the model, we start with one pair of convolutional layers and max pooling and increase it gradually. We find that two pairs can obviously provide higher accuracy than one pair, and more pairs will bring more time consumption but no more performance gain. Thus, we use two pairs of convolutional layers and max pooling finally. Some key parameters, such as the convolution window size and sliding step, are limited by the input graph size and determined by repeated trails. Moreover, we keep the model’s default values for some parameters that do not affect the performance.

Figure 4b,d show the evolution of the training/validation accuracy and loss with the epoch, when the temporal and spectral data are used, respectively. At the end of the training cycles, 96.97% and 96.78% validation accuracy values are obtained for the temporal and spectral data, accordingly. Following the training and validation processes, we use the testing datasets to evaluate the performance of the two CNN models. The confusion matrixes when using the temporal and spectral data are shown in Figure 4c,e, respectively. Of the classification values, 97.0% and 97.1% are, respectively, obtained using the CNN models of the temporal and spectral data. The results of the confusion matrixes in this contrast experiment confirm the effectiveness of the CNN models to distinguish between the “infested” and “healthy” signals.

The FalseAlarm (false infested or false positive) is a critical performance metric of the CNN models that should be decreased in our experiments, to avoid removing or applying a treatment to a healthy tree because of sensor false alarms. Given the false positives FP and the true positives TP in a confusion matrix, the FalseAlarm is expressed as FalseAlarm=FP/(TP+FP) [25]. Using the results of the confusion matrixes in Figure 4c,e, the value of the FalseAlarm is 3.64% and 3.56% for the CNN models of the temporal and spectral data, respectively. To reduce the value of the FalseAlarm, we introduce integrating the classification results of the two CNN models such that a temporal example and its corresponding spectral one are marked to be ”infested” if and only if the two CNN models produce ”infestation” classification results. In other words, if a temporal example is classified as ”infested” by the temporal CNN model, while its corresponding spectral example is classified as ”healthy” by the spectral CNN model, then we classify this overall example as ”healthy”. By adopting this approach, the sensor FalseAlarm is decreased to 2.82%. Compared with the original 3.64% and 3.56% FalseAlarm values of the temporal and spectral data, the new 2.82% FalseAlarm obtained after using the strict decision-making method has improvement percentages of 22.5% and 20.8%, respectively. Consequently, we decide to apply the introduced merged classification approach to count the infestation alarms when classifying the infested and healthy trees in the subsequent section.

## 4. Classifying Infested and Healthy Trees Using CNNs

In this section, we use the aforementioned merged classification approach with the trained CNN models to distinguish between infested and healthy trees. In other words, in an experiment involving infested and healthy trees, we record equal data examples from the individual trees and pass them to the CNN models with the merged classification approach to count the number of infestation alarms for each tree. These experiments are carried out when trees are located in a controlled environment and in an outdoor farm.

**Figure 5:**
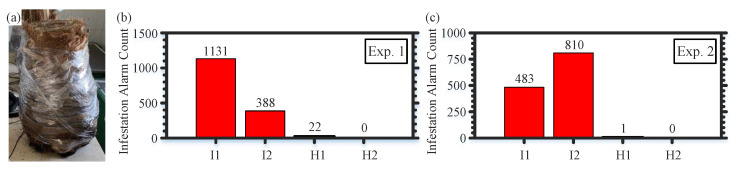
(**a**) Example of a tree used in the controlled environment experiments. Infestation alarm count produced by our sensor during Exp. 1 (**b**) and Exp. 2 (**c**). I: infested tree; H: healthy tree.

Focusing first on the controlled environment experiments where the trees are located in a closed room with windows so that the trees may be exposed to mild acoustic noise produced by birds flying around the room and/or humans inside the room. We arrange two different experiments (Exp. 1 and Exp. 2) in the controlled environment such that each experiment involves four trees (two infested and two healthy). Part of the data collected in Exp. 1 are used to train the CNN models; however, the trees and data of Exp. 2 are never included in training the CNN models. This experimental design is important for investigating the generalization of the trained CNN models. The infested trees in Exp. 1 and Exp. 2 include larvae less than three weeks old, which is controlled during the artificial infestation process [12]. The height range of the infested and healthy trees, placed in the controlled environment, is 1–1.5 m. Figure 5a shows an example of a tree used in the experiments, while the optical fiber is wrapped around it and a plastic wrap is added as an outer layer over the fiber and the tree. For each tree in Exp. 1 (Exp. 2), we record 129,761 (144,755) temporal images with identical number of their corresponding spectral images. As Figure 5b,c show, the merged classification approach is generalized and can efficiently distinguish between the infested and healthy trees in the two experiments by providing obvious contrasts in the number of alarms between the infested and healthy trees. Thus, these contrast experiments demonstrate the efficacy of the reported method for identifying the infested and healthy trees in the designed controlled environment.

**Figure 6:**
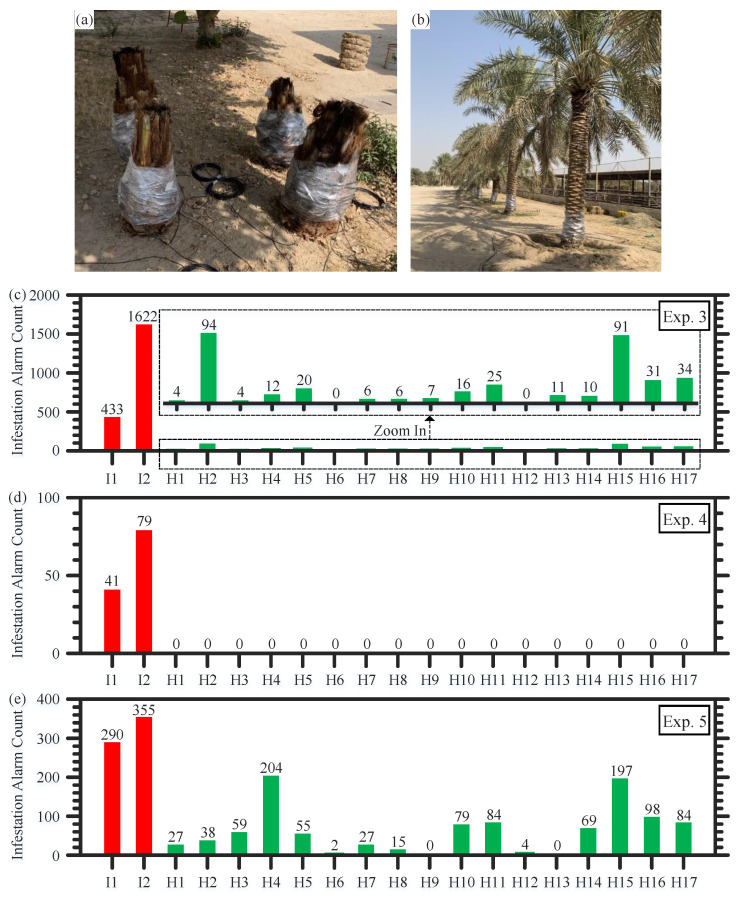
In the outdoor farm, there are two short infested and two short healthy trees (**a**), and another 15 tall typical trees (**b**). Infestation alarm count produced by our sensor during Exp. 3 (**c**), Exp. 4 (**d**), and Exp. 5 (**e**). I: infested tree; H: healthy tree.

In turn, we also conduct other experiments on an outdoor farm that includes different types of acoustic noises produced by wind, birds, farm animals, and vehicles. The experiments (Exp. 3–Exp. 5), conducted on the outdoor farm, include the same trees consisting of two artificially infested trees and 17 healthy trees, but the experiments are carried out under different wind speed ranges. The two artificially infested trees and two other healthy trees are short (1–1.5 m) (Figure 6a), while the remaining 15 healthy trees are typical and tall (Figure 6b). Again, the trees and data of Exp. 3–Exp. 5 are completely new to the CNN-trained models. For each tree in Exp. 3, we record 172,686 data examples during which the wind speed changes within a [0, 8] mph range. Considering the number of infestation alarms in Figure 6c of Exp. 3, the merged classification approach can distinguish well between the infested and healthy trees on the outdoor farm.

To investigate the impact of the wind speed on the performance of our sensor, we further carry out Exp. 4 and Exp. 5 at different wind speed ranges. In particular, Exp. 4 is carried out in the “light air” and “light breeze” conditions where 16,694 data examples per tree are recorded when the wind speed is within a [3, 5] mph range. In contrast, we collect 22,763 data examples per tree for Exp. 5 in the “gentle breeze” and “moderate breeze” conditions where the wind speed is within a [9, 14] mph range. In Exp. 4, when the wind speed is relatively low, the system performs outstandingly and perfectly discriminates between the infested and healthy trees (Figure 6d). As the wind speed increases to that range of Exp. 5, the performance of the sensing system degrades. These results are in good agreement with our findings in [15]; wind is the main source of noise in our system, compared to the acoustic noise of birds and humans that is greatly attenuated when propagating through the air before reaching the fiber [26]. Thus, these contrasting experiments conducted outdoors show that the best performance of our sensor can be obtained when wind speeds are less than 9 mph.

## 5. Discussion

In the experiments conducted in the controlled environment and outdoor farm, one can observe that the infestation alarm count for an infested tree is much lower than the tree’s total number of recorded examples. This is attributed to the fact that the larvae may not continuously produce sound and/or their sound is sometimes not strong enough to be picked up by the optical fiber. Thus, it is important to differentiate between classifying “infested” and “healthy” acoustic signals, presented in Section 3, and classifying infested and healthy trees, described in Section 4. For example, regarding the acoustic signals, it is straightforward to calculate the FalseAlarm values because the data size and class are known. However, for the real scenario of classifying the trees, the FalseAlarm cannot be calculated because even an infested tree produces “infested” and “healthy” signals. Thus, we decide to rely on counting the infestation alarms to distinguish between the healthy and infested trees. Considering the practical application of the sensor, we can select few healthy trees as references and based on their maximum infestation false alarm count, we can set an appropriate threshold for infestation alarm count to announce a tree as infested.

We also compare our optical fiber DAS and CNN method with existing RPW detection technologies. Table 1 summarizes the comparison results. We can observe that the acoustic detection methods have attracted the most research interest for RPW detection in past years. Among all methods

Based on acoustic sensors, our technique based on DAS with CNN algorithm demonstrates advantages in most aspects of concern, including high detection accuracy, 24/7 monitoring, unattended, early detection capability, low cost for large-scale applications, and moderate computational complexity. However, our sensor suffers from the degradation in performance outdoors at high wind speeds, which will require further investigation and improvement. Thus, we believe that our DAS-based method is worthy of implementation in large-scale practical applications.

To sum up, this work aims to use optical fiber DAS to monitor RPW infestation in outdoor date plantations. The acoustic data recorded by the optical fiber DAS are passed to a trained CNN model to decide whether the acoustic signal is “infested” or “healthy”. For each tree, the infestation alarm count produced by the CNN model can be used to decide whether the tree is infested or healthy. The significance of this work is to pave the way for future experiments, as we plan to use our sensor to detect RPW in naturally infested trees. However, this may require challenging arrangements as it is difficult to find a tree at an early stage of infestation because the tree only shows signs of visual distress at a very advanced stage of infestation. In addition, we will consider improving the overall performance of the CNN model by training it further on diverse data to improve the performance of our system in terms of the contrast between the infestation alarm counts of the infested and healthy trees.

## 6. Conclusions

We report on the integration of optical fiber DAS and CNN for the early detection of RPW in large farms. The temporal and spectral acoustic signals recorded by the optical fiber DAS are used to train CNN models, resulting in classifying the “infested” and “healthy” signals with accuracy values of 97.0% and 97.1%, respectively. Merging the classification results of the temporal and spectral CNN models can reduce the FalseAlarm performance metric of the sensor by ∼20%. Our sensor shows success in recognizing the infested and healthy trees in a controlled environment and an outdoor farm, with a high efficiency when the wind speeds are less than 9 mph outdoors. The main advantage of the reported sensor, compared to other current technologies, is that the sensor can provide 24/7 monitoring while offering wide coverage of the farming area, using only a single optical fiber cable. In contrast, the performance of the reported sensor still requires improvement when working outdoor at high wind speeds.

## Figures and Tables

**Table 1:** Comparison of our DAS+CNN method with existing sensors for RPW detection, in chronological order.

Method	Processing Technique	Invasive or Not	Performance or Accuracy	Advantages (Disadvantages)
An acoustic sensor (commercial piezoelectric microphone), 2008 [27]	Speech recognition method, vector quantization (VQ), and Gaussian mixture modeling (GMM)	Not	98% accuracy	Automatic detection using simple commercial hardware (A sound-isolated box is used)
An acoustic sensor (Piezoelectric sensor), 2009 [28]	Feature extraction, GMM	Invasive	99.1% accuracy	Automatic detection with well-designed algorithms (High computational complexity)
An acoustic sensor (electronic device with acoustic probe), 2010 [29]	FFT, studying the sound intensity around 2250 Hz	Invasive	The infested sound intensity increases around 1 dB from −20 dB	Detection of a small number of larvae with a simple signal processing method (Low contrast between infested and non-infested sound)
An acoustic device (acoustic probe and headphone set), 2010 [14]	Bandpass filtering, amplification	Invasive	97% accuracy	Simple and portable hardware (Manual identification with four detection positions needed)
A radiography system (X-ray technology), 2012 [30]	Visual detection based on X-ray photos	Not	Observable larvae on the photos	Simple and visual operation (Difficult for large-scale applications)
An acoustic sensor (audio probe), 2013 [13]	Filtering and amplification, feature vector quantization	Invasive	90% accuracy	Autonomous and continuous detection with explicit audio analysis algorithm (Extensive field experiments are needed in the future)
Thermal imaging (infrared thermal camera), 2015 [11]	Thermal infrared images (TIR), leaf temperature maps, canopy representative temperature, crop water stress index (CWSI)	Not	Less than 75% accuracy	Large-scale and non-invasive detection (Susceptible to environmental conditions)
An acoustic sensor (piezoelectric microphone), 2016 [31]	Likelihood indication by observer, speech recognition algorithm same as that in Ref. [27]	Not	75% accuracy by humans, 80% accuracy by machine	Manual and automated detection are compared (Susceptible to wind)
Some optical devices (digital camera, thermal camera, TreeRadarUnit (Radar 2000, Radar 900), resistograph, magnetic DNA biosensor, and near-infrared spectroscopy (NIRS)), 2020 [32]	Visual analysis, the analysis of variance (ANOVA) PROC GLM procedure, response of the leaf spectral absorbance	Not	Accuracy: visual approach 87%, Radar 2000 77%, Radar 900 73%, resistograph 73%, thermal camera 61%, digital camera 52%, and magnetic DNA 63%	All used methods are non-invasive with a detailed comparison (Accuracy needs to be further improved)
An IoT system (commercial accelerometer sensor), 2020 [33]	FFT, the estimation of power spectral density (PSD), peaks average difference (PAD) analysis	Invasive	Observable signature of the infestation	Simple hardware with a connection to network (Low sensitivity and contrast)
An acoustic sensor (USB microphone), 2021 [34]	Feature extraction using Mel Frequency Cepstrum Coefficient (MFCC), discrete Fourier transform (DFT), artificial neural network (ANN), Alexnet-convolutional neural networks (CNN)	Not	99.2% accuracy	Simple hardware and concise algorithm (A plastic tube is used to imitate the real tree)
A large-scale imaging detection method (aerial and street view), 2021 [35]	CNN, faster R-CNN ResNet-50 FPN, XResNet,	Not	Aerial and street images can be mapped to actual palm trees	Automatic large-scale detection (Limited number of infested palm tree images available online)
An IoT system (acoustic detection of the public TreeVibes dataset), 2021 [36]	Modified mixed depthwise CNN (MixConvNet)	Invasive	95.90% accuracy	Integration in a smartphone application with advanced algorithm (Only verified on the public TreeVibes dataset)
An optical fiber distributed acoustic sensor (ours)	CNN	Not	Around 97.0% accuracy	Provides 24/7 monitoring on large-scale farms (Low performance at high wind speeds)

## Data Availability

The data presented in this study are available on reasonable request from the corresponding author. The data are not publicly available due to privacy.
